# Friend retrovirus drives cytotoxic effectors through Toll-like receptor 3

**DOI:** 10.1186/s12977-014-0126-4

**Published:** 2014-12-24

**Authors:** Kathrin Gibbert, Sandra Francois, Anna M Sigmund, Michael S Harper, Bradley S Barrett, Carsten J Kirchning, Mengji Lu, Mario L Santiago, Ulf Dittmer

**Affiliations:** Institute for Virology of the University Hospital in Essen, University of Duisburg-Essen, Essen, Germany; Institute for Medical Microbiology of the University Hospital in Essen, University of Duisburg-Essen, Essen, Germany; Department of Medicine, University of Colorado Denver, Aurora, CO 80045 USA

**Keywords:** Toll-like receptor 3, Friend Retrovirus, Dendritic cells, NK cells, Cytotoxic T cells, Pathogen recognition

## Abstract

**Background:**

Pathogen recognition drives host defense towards viral infections. Specific groups rather than single members of the protein family of pattern recognition receptors (PRRs) such as membrane spanning Toll-like receptors (TLRs) and cytosolic helicases might mediate sensing of replication intermediates of a specific virus species. TLR7 mediates host sensing of retroviruses and could significantly influence retrovirus-specific antibody responses. However, the origin of efficient cell-mediated immunity towards retroviruses is unknown. Double-stranded RNA intermediates produced during retroviral replication are good candidates for immune stimulatory viral products. Thus, we considered TLR3 as primer of cell-mediated immunity against retroviruses *in vivo*.

**Results:**

Infection of mice deficient in TLR3 (TLR3^−/−^) with Friend retrovirus (FV) complex revealed higher viral loads during acute retroviral infection compared to wild type mice. TLR3^−/−^ mice exhibited significantly lower expression levels of type I interferons (IFNs) and IFN-stimulated genes like *Pkr* or *Ifi44*, as well as reduced numbers of activated myeloid dendritic cells (DCs) (CD86^+^ and MHC-II^+^). DCs generated from FV-infected TLR3^−/−^ mice were less capable of priming virus-specific CD8^+^ T cell proliferation. Moreover, cytotoxicity of natural killer (NK) cells as well as CD8^+^ T cells were reduced *in vitro* and *in vivo*, respectively, in FV-infected TLR3^-/-^ mice.

**Conclusions:**

TLR3 mediates antiretroviral cytotoxic NK cell and CD8^+^ T cell activity *in vivo*. Our findings qualify TLR3 as target of immune therapy against retroviral infections.

## Background

The host immune system offers a variety of different innate receptors to sense invading viruses. These pattern recognition receptors (PRR) recognize pathogen-associated molecular pattern (PAMPs), which are highly conserved molecular structures produced by pathogens but not by the host cells. One important family of PRRs includes the Toll-like receptors (TLRs) that span cell or endosomal membranes. The latter include TLR3, TLR7/8 and TLR9 which recognize double-stranded RNA (dsRNA), single-stranded RNA or CpG-containing DNA, respectively (reviewed in [1]). Cytosolic PRRs such as cyclic GMP-AMP synthase (cGAS), retinoic acid inducible gene I (RIG-I) and Melanoma Differentiation-Associated protein 5 (MDA5) also sense replication intermediates of numerous viruses [2-4]. Virus sensing PRRs principally mediate induction of type I interferon (IFN) production and thus expression of interferon-stimulated genes to elicit an antiviral state in the cell. Many viruses have developed mechanisms to evade their host sensors resulting in impaired host immunity (reviewed in [5]). Redundancy in virus recognition, namely employment of a specific set of multiple PRRs, prevents viral escape from host surveillance.

Studies *in vitro* revealed that multiple PRRs such as DC-SIGN, TLR7/8, TLR9, cGAS and TRIM5α sense retroviruses including HIV and SIV (reviewed in [2,6-10]). However, *in vivo* studies determining their impact in directing antiretroviral innate and adaptive immune responses remains limited. Using knockout mice, myeloid differentiation primary response 88 (MyD88) and TLR7-dependent viral sensing were previously shown as critical for the development of neutralizing antibody responses against murine leukemia virus (MuLV) infections [11,12]. However, the induction of T cell responses only partially depended on the MyD88-TLR7 pathway [11,12]. The impact of innate sensing mechanisms on Natural Killer (NK) cell responses, which are also critical for antiretroviral immunity [13,14], remains unknown.

In this report, we investigated whether the dsRNA sensor TLR3 [15] is involved in retrovirus sensing. Duplex formation of retroviral RNA prior to reverse transcription may occur during retroviral life cycle [16] and higher order dsRNA structures like stem loops located in retroviral long-terminal repeats were described [17,18]. To investigate the impact of TLR3 during retroviral infections *in vivo*, we used the Friend Retrovirus (FV) mouse model. The FV complex is comprised of two retroviruses: the replication-competent helper virus called Friend murine leukemia virus (F-MuLV), which is non-pathogenic in adult mice, and the replication-defective, pathogenic spleen focus-forming virus (SFFV) [19]. FV induces erythroleukemia in susceptible mice. In contrast, resistant strains, such as the C57BL/6 mice that were used in the current study, mount potent immune responses during acute infection to recover from disease [20]. Our findings provide evidence that TLR3 mediates retroviruses sensing which impacts early cellular antiretroviral immune response *in vivo*.

## Results

### Increased viremia in TLR3^−/−^ mice

**Figure 1:**
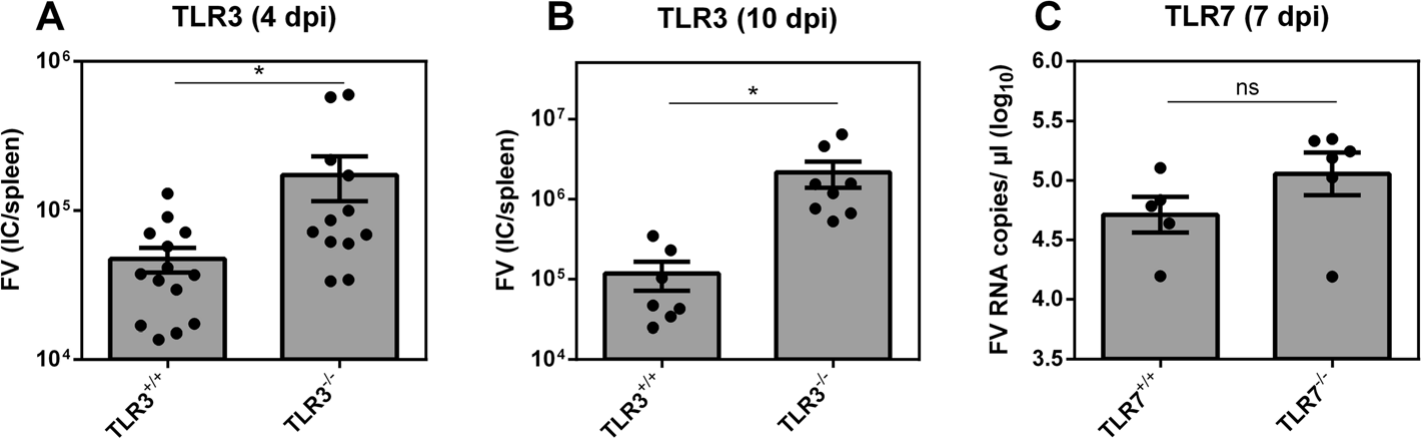
**Viral loads in FV-infected TLR3** ^**−/−**^ **and TLR7** ^**−/−**^ **mice.** TLR3^+/+^ and TLR3^−/−^ mice were infected with 20,000 SFFU of FV and viral loads in the spleen were determined by infectious center assay at days 4 **(A)** and 10 **(B)** post infection. In addition, plasma viral loads from FV-infected TLR7^−/−^ and TLR7^+/+^ mice at 7 dpi were measured by qPCR **(C)**. At least five mice per group were analyzed. Individual percentages and mean values are indicated by bars and dots + SEM. Statistically significant differences between FV-infected TLR3^+/+^ and TLR3^−/−^ mice are indicated by * for p < 0.05.

Deletion of MyD88 in mice did not result in a significant increase in acute FV viremia [11,12], suggesting the existence of alternative sensing pathways. Since TLR3 is activated through TIR-domain-containing adapter-inducing interferon-β (TRIF) [21-23], we tested if TLR3 is involved in immune recognition of retroviruses. Thus, we infected wild type mice (TLR3^+/+^) and mice deficient in TLR3 (TLR3^−/−^) with FV and analyzed the viral loads at days 4 and 10 post infection. At both time points, a significant increase in viral loads was detected in TLR3^−/−^ mice (Figure 1). At the earlier time point the mean viral burden was up to 4 times higher in TLR3^−/−^ compared to wild type mice (Figure 1A) and the effect was even stronger at 10 dpi (>18-fold; Figure 1B). In contrast, TLR7 deficiency in mice did not impact acute FV infection (Figure 1C; 7 dpi). These data indicate that TLR3 sensing contributes to immune control of FV during the acute phase of infection.

### Impaired DC activation in FV-infected TLR3^−/−^ mice

**Figure 2:**
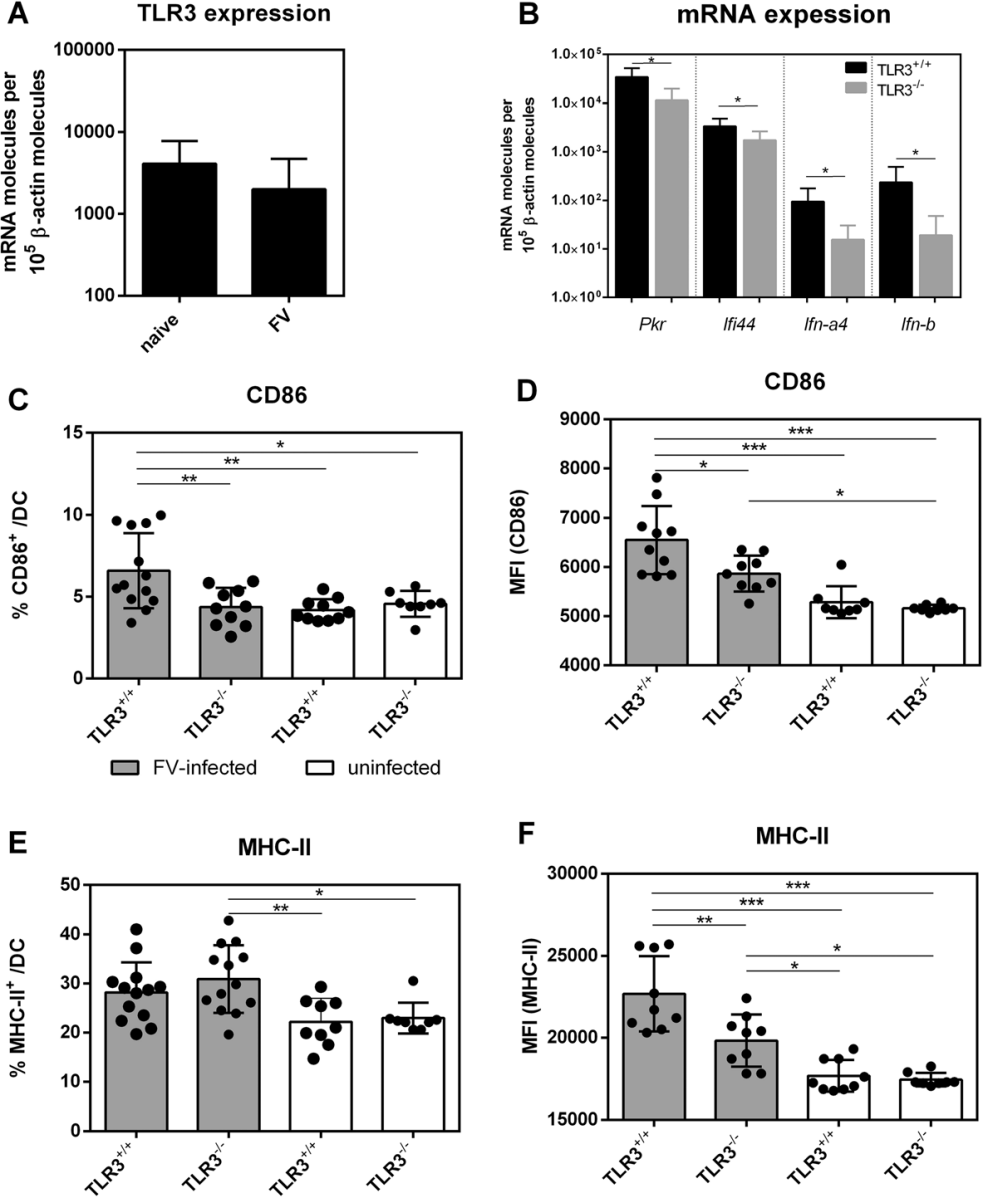
**Phenotypic analysis of splenic DCs in FV-infected mice.** TLR3^+/+^ and TLR3^−/−^ mice were infected with 20,000 SFFU of FV. At 4 dpi splenic DCs were isolated and analyzed for *Tlr3* mRNA expression **(A)** and mRNA expression of *Pkr, Ifi44, Ifn-a4* and *Ifn-b* **(B)**. Splenic DCs (CD11c^+^ CD11b^+^) were also analyzed by flow cytometry. Surface expression of the costimulatory molecule CD86 **(C, D)** and MHC-II **(E, F)** were determined. Uninfected TLR3^+/+^ and TLR3^−/−^ mice were used as controls (white bars). A minimum of nine mice per FV-infected group were analyzed and the individual values are indicated by bars and dots + SEM. Experiments were repeated at least twice. Statistically significant differences between the groups are indicated by * for p < 0.05, ** for p < 0.01 and *** for p < 0.001.

As TLR3 is mainly expressed by myeloid DCs (mDCs) and macrophages (reviewed in [24,25]), but not plasmacytoid DCs or T cells, we analyzed the impact of TLR3 sensing on the activation and maturation of mDCs and macrophages during acute FV infection (4 dpi). Firstly, we measured if the expression level of this receptor is up-regulated in mDCs during FV infection. As depicted in Figure 2A, *Tlr3* mRNA was detectable in mDCs of both uninfected, as well as FV-infected mice, whereas the relative expression levels did not differ significantly. As downstream signaling of TLR3 leads to the induction of type I IFN and subsequent expression of interferon-stimulated genes (ISG), we analyzed antiviral gene expression during acute FV infection in TLR3^−/−^ and TLR3^+/+^ mice. Therefore, we isolated total mRNA from mDCs of FV-infected TLR3^−/−^ and TLR3^+/+^ mice and analyzed the expression of specific ISGs (*Pkr, Ifi44*) as well as the early type I IFNs (*Ifn-a4, Ifn-b*). Expression levels of all of the 4 mRNAs analyzed were increased during FV-infection compared to uninfected controls (data not shown), however mDCs of FV-infected TLR3^−/−^ mice had reduced expression levels in comparison to those of infected TLR3^+/+^ mice (Figure 2B). The overall expression level of type I IFNs during FV infection in wild type mice was quite low which is in line with previous data from our group [26,27]. As the total numbers of mDCs in the spleen did not vary significantly in FV-infected TLR3^+/+^ and TLR3^−/−^ mice (data not shown), we next analyzed the activation of mDCs which was measured by the surface expression of the costimulatory molecule CD86 and major histocompatibility complex class II (MHC-II). Although the overall activation of mDCs during FV infection was low, mDCs were significantly higher activated during FV infection than in uninfected control mice (TLR3^−/−^ or TLR3^+/+^ mice). In FV-infected mice a significantly reduced percentage of CD86^+^ mDCs was detected in TLR3^−/−^ mice in comparison to TLR3^+/+^ mice (Figure 2C) and a reduced mean fluorescence intensity of CD86 on mDCs of FV-infected TLR3^−/−^ mice was found (Figure 2D). While infected TLR3^−/−^ mice did not have lower percentages of MHC-II^+^ mDCs compared to wild type mice (Figure 2E), the surface expression levels of MHC-II on these cells were significantly decreased in FV-infected TLR3^−/−^ mice (Figure 2F). In contrast, the absolute numbers of macrophages in the spleen of FV-infected wild type and TLR3^−/−^ mice, as well as their activation status, did not differ (data not shown). Thus, TLR3 enhanced expression of type I IFNs and antiviral ISGs as well as activation of mDCs upon FV infection.

### NK cell cytotoxicity depends on TLR3

**Figure 3:**
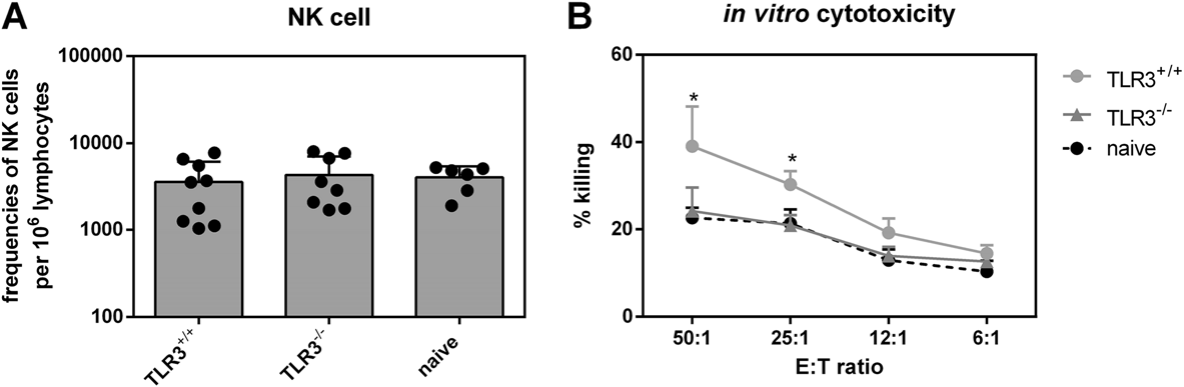
**Effector functions of NK cells.** TLR3^+/+^ and TLR3^−/−^ mice were infected with 20,000 SFFU of FV. At 4 dpi numbers of NK cells (CD3^−^ CD49b^+^ NK1.1^+^) in the spleen of FV-infected or naive mice were analyzed by flow cytometry **(A)**. At least six mice of minimum two independent experiments were used and the mean values are shown by bars and dots + SEM. The cytotoxic potential of splenic NK cells was analyzed in an *in vitro* NK cell cytotoxicity assay. NK cells were isolated from spleens of FV-infected TLR3^+/+^ and TLR3^−/−^ mice **(B)**. FV-derived tumor cells (FBL-3) were stained with CFSE and co-cultured with isolated NK cells at different effector-target ratio of 50:1 to 6:1 for 24 h. At least four mice were used for the analysis. Statistically significant differences between the groups of infected TLR3^+/+^ and TLR3^−/−^ mice are indicated by * for p < 0.05.

Since mDCs bridge pathogen recognition and immune responses, we investigated effector functions of specific immune cell populations. As TLR3 already had an impact on viral loads at 4 dpi (Figure 1A), we analyzed the numbers and effector functions of NK cells at this early time point [14]. We did not find an increase in total numbers of NK cells (NK1.1^+^ CD49b^+^ CD3^−^) or percentages of activated (CD69) NK cells in the spleen of FV-infected mice compared to uninfected control mice (Figure 3A, and data not shown). Moreover, no significant difference in NK cell activation was observed between FV-infected TLR3^−/−^ and wild type mice (Figure 3A). However, using an *in vitro* cytotoxicity assay, we observed a significant decrease in killing of FV-derived tumor cells (FBL-3) by NK cells isolated from FV-infected TLR3^−/−^ mice in contrast to NK cells from wild type mice (Figure 3B). NK cells from FV-infected wild type mice were twice as effective in killing target cells as those from TLR3^−/−^ mice. Their killing capacity was very low, at comparable levels of NK cells from naive control mice. Thus, TLR3 is required for cytotoxic NK cell responses during acute FV infection.

### Impaired T cell responses in TLR3-deficient FV-infected mice

**Figure 4:**
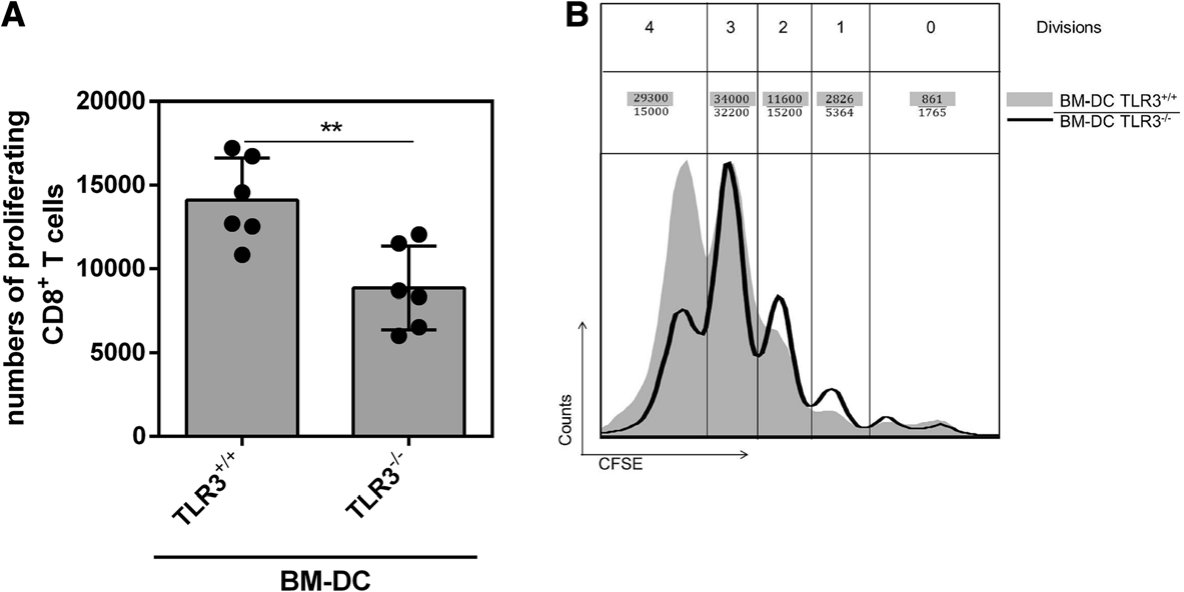
**Proliferation of FV-specific CD8** ^**+**^ **T cells** ***in vitro*** **.** TLR3^+/+^ and TLR3^−/−^ mice were infected with 20,000 SFFU of FV. At 4 dpi bone marrow cells were isolated and cultured for 7 days to obtain BM-DCs. BM-DCs were loaded with FV GagL peptide and co-cultured with CFSE-labeled FV-specific CD8^+^ T cells for 3 days. Proliferation of transgenic CD8^+^ T cells was determined by flow cytometry by loss of CFSE dye as calculated numbers of proliferating cells **(A)** or a representative histogram showing the amount of cells in each generation **(B)**. A minimum of six mice were used for analysis. At least two independent experiments were performed. Significant differences between the groups are indicated by * for p < 0.05.

We next investigated T cell responses at 10 days post infection, when viral loads in TLR3^−/−^ mice were prominently higher than in wild type mice (Figure 1B). Firstly, we analyzed if DCs from FV-infected TLR3^−/−^ mice had an altered capacity to stimulate CD8^+^ T cell proliferation *in vitro.* We generated bone marrow (BM) derived DCs isolated from FV-infected TLR3^−/−^ and TLR3^+/+^ mice and loaded these with a FV-specific CD8^+^ T cell epitope peptide. Afterwards, peptide-loaded BM-DCs were co-cultured with CFSE-labeled FV-specific TCR transgenic CD8^+^ T cells and T cell proliferation was measured. As shown in Figure 4A, T cell stimulation with BM-DCs generated from TLR3^−/−^ mice resulted in lower numbers of proliferating CD8^+^ T cells (fewer CD8^+^ T cell proliferation cycles) than those of wild type mice. This decrease was also seen in a representative histogram in Figure 4B showing that the proportion of cells particularly in the fourth daughter population (left peak; generation 4) was strongly reduced in cultures with BM-DCs generated from TLR3^−/−^ mice. The data implicate TLR3 in priming of FV-specific CD8^+^ T cells.

**Figure 5:**
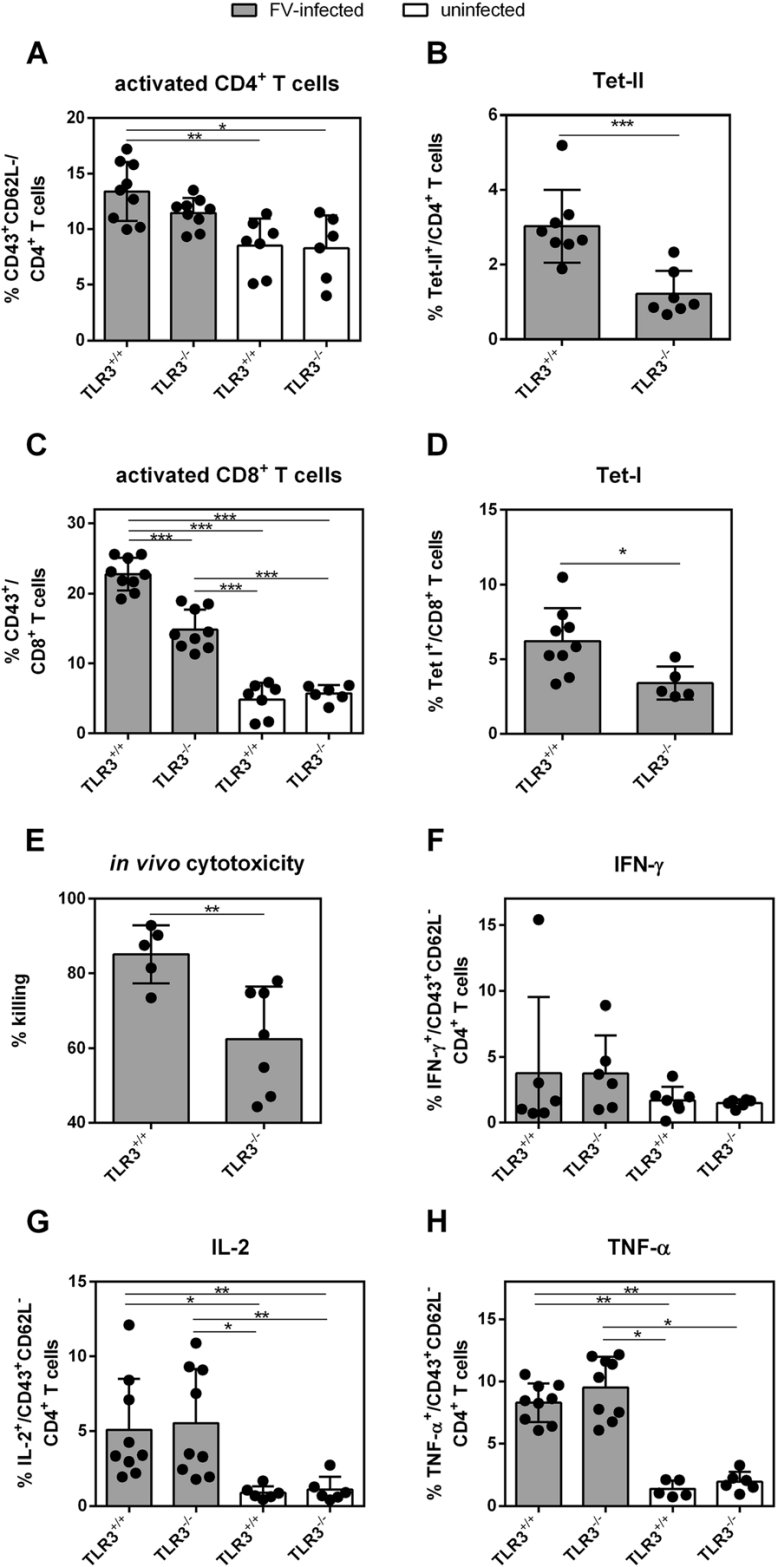
**FV-specific CD4** ^**+**^ **and CD8** ^**+**^ **T cell responses in FV-infected TLR3** ^**+/+**^ **and TLR3** ^**−/−**^ **mice.** TLR3^+/+^ and TLR3^−/−^ mice were infected with 20,000 SFFU of FV. CD4^+^ and CD8^+^ T cells were analyzed at 10 dpi by flow cytometry. Percentages of activated (CD43^+^ CD62L^−^, **A)** CD4^+^ T cells were determined. For the analysis of virus-specific CD4^+^ T cells **(B)** splenocytes were stained with MHC class II-antibody tetramers specific for F-MuLV env fn20. To analyze the function of CD4^+^ T cells, the intracellular expression of IFN-γ **(C)**, IL-2 **(D)** and TNF-α **(E)** was measured in activated effector CD4^+^ T cells. Percentages of activated (CD43^+^, **F)** CD8^+^ T cells and virus-specific effector CD8^+^ T cells **(G)**, which are specific for the FV GagL epitope stained for GagL class I tetramers, were analyzed by flow cytometry. Uninfected TLR3^+/+^ and TLR3^−/−^ mice were used as controls (white bars). At least five mice per group were analyzed and the mean value for each group is indicated by bars and dots. At least 2 independent experiments were performed. Statistical differences between the groups are indicated by * for p < 0.05, ** for p < 0.01 and *** for p < 0.001. **(H)** Splenocytes from naive mice were loaded with the FV-specific DbGagL peptide and labeled with CFSE. Target cells were injected intravenously into naive or FV-infected TLR3^+/+^ and TLR3^−/−^ mice. Two hours after transfer, donor cells from spleen were analyzed. The figure shows the percentage of target cell killing in the spleen. Two independent experiments with three mice per group were performed and mean values are shown by bars. Differences between both groups are indicated by ** for p < 0.01.

To monitor effector T cell responses during acute FV infection in TLR3-deficient and wild type mice, we infected these mice with 20,000 SFFU of FV. At 10 dpi, splenocytes were analyzed for CD4^+^ and CD8^+^ T cell responses. We did not detect a difference in the overall percentage of activated (CD43^+^ CD62L^−^) CD4^+^ T cells (Figure 5A). However, significantly lower numbers of virus-specific CD4^+^ T cells were detected in FV-infected TLR3^−/−^ mice (Figure 5B). We observed no difference in the expression of IFN-γ (Figure 5C), IL-2 (Figure 5D) or TNF-α (Figure 5E) indicating that CD4^+^ T cell functions did not depend on TLR3 during acute FV infection. Next, we analyzed CD8^+^ T cells, which are key players in anti-FV immunity [28,29]. In comparison to TLR3-deficient mice, wild type mice had significantly higher percentages of activated (CD43^+^, Figure 5F) as well as virus-specific CD8^+^ T cells measured by tetramer staining (Figure 5G). To address if the impaired activation of the CD8^+^ T cells correlated with reduced cytotoxic effector functions and consequently higher viral loads (Figure 1), we performed an *in vivo* CTL assay. We observed a significantly higher killing of FV peptide-labeled target cells in wild type mice (mean: 85%) in contrast to TLR3^−/−^ mice (mean: 62.5%; Figure 5H). These results demonstrate that TLR3 is essential for the induction of potent cytotoxic CD8^+^ T cell effector functions during acute FV infection.

## Discussion

Efficient sensing by the innate immune system is the first step towards an effective antiviral immune response. In an effort to determine which innate sensing pathways are critical for retrovirus infections *in vivo*, mice deficient in various innate sensors were infected with murine retroviruses such as MuLV. Previously, it was shown that TLR7 and MyD88 were required for a potent antiretroviral humoral immune response [11,12]. However, T cell responses were only partially affected by MyD88 signaling, and no data exists on sensors required for NK cell activity. Here, we demonstrate for the first time that TLR3 sensing is involved in cytotoxic T cell and NK cell responses during acute FV infection.

Interestingly, no differences in viral loads were observed between wild type and MyD88-deficient mice during acute infection (up to 1 week post infection) [11]. Our data showing that TLR7 does not impact acute FV infection (see Figure 1C) is consistent with this finding, but contrasts recent data from the same group [30]. We hypothesize that the lack or inconsistent impact of TLR7/MyD88 on acute FV infection may reflect the fact that the neutralizing antibody responses do not play a significant role in inhibiting FV at the earliest infection time points [31]. Notably, at later time points (starting 2 weeks post infection) viral loads were increased in MyD88^−/−^ mice, consistent with a weakened humoral immune response. We recently provided evidence that NK cell responses could significantly inhibit acute FV replication *in vivo* [14]. Thus, our findings showing enhanced acute FV replication in TLR3^−/−^ mice are consistent with a strong impact of TLR3 on cytotoxic NK cell responses. TLR3 is strongly expressed by mDCs, whereas its expression is weak in murine B cell subsets [32]. In contrast, TLR7 is highly expressed by follicular B, marginal zone B, Peyer´s patch B and B-1B cells and the expression level in mDCs is rather low [32]. This may explain that TLR7 is required for efficient antibody responses [11,12] as it might directly influence B cell responses and not NK or T cell responses.

Other known innate sensors for retroviral infections are cGAS [2], DC-Sign [6], TLR9 [33] and zinc-finger antiviral protein [34]. They were all observed to be important for induction of type I IFN by retroviruses, but their influence on cellular or humoral immune responses has not been investigated so far. Antiviral immune responses can be affected by many sensors, which were described for other virus infections. This depends on the host cell type and on the time point of the infection. Especially TLRs are not ubiquitously expressed, but rather by specific immune cells like mDCs, pDCs, macrophages, B cells and others [35-37]. Other PRRs like MDA5 or Rig-I are found in the cytosol of almost every cell type making them efficient general sensors for viral infections. For influenza virus infection various PRRs were shown to be required for efficient induction of anti-viral immune responses. Influenza virus infection is sensed by many different PRRs (TLR3, TLR7 and Rig-I) (reviewed in [38]), and they all have distinct influences on the cellular and humoral immune responses against the virus. It was shown that TLR3, TLR7 and MyD88 signaling is not required for efficient T responses during influenza infection [39,40], but both TLR7 and MyD88 are critical for B cell responses during influenza infection [40]. Koyama and colleagues investigated that Rig-I is also not needed for a potent CD8^+^ T cell response, whereas B cells and CD4^+^ T cells require MyD88 and Rig-I [39]. West Nile virus (WNV) vaccination studies revealed that MyD88 and TLR3 are both required for efficient humoral immune responses [41], and during a WNV infection Rig-I and MDA5 [42-44], as well as TLR3 and TLR7 [45,46] are involved in immune recognition. Infection with Theiler`s murine encephalomyelitis virus requires sensing of both TLR3 and MDA5 [47,48]. Tabeta and colleagues reported that during mouse cytomegalovirus infection deficiency of TLR3 and TLR9 increases the infection due to reduced type I IFN secretion and NK cell activation [49]. This demonstrates that in many viral infections immune responses are initiated by various sensors and distinct PRRs have unique roles during viral defense.

Toll-like receptor 3 recognizes double-stranded RNA during viral infections [15]. Retroviruses consist of two single-stranded RNA strands which are entwined within the core. Together with the viral proteins they build a dimeric RNA complex [50]. These RNA strands form high order secondary structures like stem loops which might be targeted by TLR3 [17,18,51-53]. A recent study could show that TLR3 recognizes stem structures in single-stranded viral RNA [54] which indicates that TLR3 is a potential immune sensor of retroviruses.

In an earlier study we have used a synthetic ligand for TLR3 (polyinosinic:polycytidylic acid, poly I:C) to treat mice during acute FV infection [55]. Stimulation of TLR3 resulted in a significant reduction in viral loads and prevented virus-induced splenomegaly as well as the onset of lethal erythroleukemia. We showed that CD8^+^ T cell responses and especially their cytotoxicity (CD107a, GzmB expression) were improved by triggering TLR3 [55]. However, TLR3 stimulation alone can still not mediate complete viral clearance in retroviral infections leading to the development of chronic infections.

Others demonstrated that stimulation of macrophages with poly I:C reduces HIV-1 infection *in vitro* [56-59] which depends on the expression of microRNA-155 [60]. Lentiviral vectors [61] as well as *in vitro* transcribed HIV-1 gag mRNA [62] were shown to be sensed by TLR3. Another interesting observation reported that a common polymorphism in the human TLR3 mediates protection from HIV-1 by increased activation PBMCs and the production of the proinflammatory cytokines IL-6 and CCL-3 [63]. This indicates that targeting TLR3 during retroviral infections might improve host immune response and thus reduce viral loads which makes TLR3 as a potential target for antiretroviral immunotherapies.

## Conclusion

In summary, we used TLR3^−/−^ mice to investigate the role of TLR3 in antiretroviral immunity utilizing the Friend retrovirus mouse model. Viral loads were significantly increased in these mice revealing that TLR3 participates in anti-FV immunity during acute infection. Specifically the cytotoxicity of NK cells and CD8^+^ T cells was significantly impaired in TLR3^−/−^ mice as compared to those of wild type controls. Triggering TLR3 activation might stimulate cytotoxic effector cells to eliminate virus-infected cells and thus be of interest to treat retroviral infections.

## Methods

### Mice

Seven to nine weeks old female C57BL/6 mice (TLR3^+/+^, TLR7^+/+^, Charles River Laboratories, Germany) were used for the experiments. Experiments were also done with TLR3^−/−^ mice [15] and TLR7^−/−^ [64] (Jackson Laboratories, USA) backcrossed more than 10 times on C57BL/6 background. All mice were treated in accordance with the regulations and guidelines of the institutional animal care and use committee of the University of Duisburg-Essen.

### Virus and viral infection

The FV stock used in these experiments was FV complex containing B-tropic Friend murine leukemia helper virus and polycythemia-inducing spleen focus-forming virus. The stock was prepared as a 15% spleen cell homogenate from BALB/c mice infected 14 days previously with 3000 spleen focus-forming units (SFFU). Mice were injected intravenously with 0.1 ml phosphate-buffered saline containing 20,000 SFFU of FV. The virus stock did not contain lactate dehydrogenase-elevating virus.

### Detection of FV-infected cells

Infectious centers (IC) were detected by 10-fold dilutions of single-cell suspensions onto *Mus dunnis* cells. Cultures were incubated for 3 days, fixed with ethanol, stained with F-MuLV envelope-specific monoclonal antibody 720 and developed with peroxidase-conjugated goat anti-mouse antibody and aminoethylcarbazol to detect foci [65].

### Plasma viral load quantification

FV plasma viral RNA loads were measured by real-time PCR as previously described [66].

### RNA isolation

Splenic DCs were separated by MACS technology (Miltenyi Biotec) and their total RNA was isolated using TRIzol reagent (Life Technologies) and Pure Link RNA Micro Kit (Life Technologies). Isolated RNA was dissolved in RNase-free water and stored at −80°C.

### Real time-PCR

Real time-PCR analysis for the quantification of *Pkr, Ifn-a4, Ifn-b, Ifi44* and *Tlr3* mRNA was performed using Power SYBR Green RT-PCR kit (Life Technologies) and Quanti Tect Primer assays (Qiagen). Primer sequences (Biomers) for *β-actin* were as follows: 5’-aaatcgtgcgtgacatcaaa-3’, 5’-caagaaggaaggctggaaaa-3’. The quantitative mRNA levels were performed by using StepOne Software v2.3 (Life Technologies) and were normalized to *β-actin* mRNA expression levels.

### Generation and culture of myeloid DCs from bone marrow cells and antigen-specific T cell proliferation

Bone marrow-derived DCs were generated as described before [67]. BM-DCs from 4 dpi infected TLR3^+/+^ and TLR3^−/−^ mice were left untreated or loaded with 0.1 μg/mL FV antigen (FV GagL peptide (85–93)). Antigen-specific CD8^+^ T cells were isolated from TCRtg mice by MACS technology (Miltenyi Biotec) and stained with CellTrace™ CFSE (Life Technologies). Afterwards, 2.5×10^5^ FV-specific CD8^+^ T cells were co-cultured with 0.5×10^5^ BM-DCs. After 3 days, proliferation of CD8^+^ T cells was assessed by flow cytometry as loss of CellTrace™ CFSE dye. Numbers of proliferating CD8^+^ T cells were calculated by the following formula:

$$ \mathbf{n}\ \left(\mathbf{proliferating}\ \mathbf{cells}\right)=\frac{\mathbf{n}\ \left(\mathbf{G}1\right)}{2}+\frac{\mathbf{n}\ \left(\mathbf{G}2\right)}{4}+\frac{\mathbf{n}\ \left(\mathbf{G}3\right)}{8} + \frac{\mathbf{n}\ \left(\mathbf{G}4\right)}{16} $$

### Cell surface and intracellular staining by flow cytometry

Cell surface staining was performed using the following antibodies: anti-CD11b (M1/70, Miltenyi Biotec), anti-CD11c (N418, BioLegend) anti-CD3 (17A2, eBioscience), anti-CD49b (DX5, eBioscience), anti-CD69 (H1.2 F3, eBioscience), anti-CD86 (GL-1, BioLegend), anti-MHC-II (M5/114.15.2, Miltenyi Biotec), anti-NK1.1 (PK136, BD Bioscience). Dead cells were excluded from analysis (positive for fixable viability dye, eBioscience). For detection of activated T cells splenocytes were stained with anti-CD4 (GK1.5, eBioscience), anti-CD8 (53–6.7, eBioscience), anti-CD43 (1B11, BioLegend) and anti-CD62L (MEL-14, eBioscience). Intracellular IFN-γ (XMG1.2, Miltenyi Biotec), IL-2 (JES6-5H4, Miltenyi Biotec) and TNF-α (MP6-XT22, BioLegend) staining was performed as described [28,29]. Data were acquired on LSR II flow cytometer (BD Bioscience) and analyses were performed using FACSDiva (BD Bioscience) and Flow Jo (Tree Star, USA) software.

### Tetramers and tetramer staining

Tetramer stainings were performed as described previously [13].

### *In vitro* NK cell cytotoxicity assay

*In vitro* NK cell cytotoxicity assay was performed using 1 × 10^4^ CFSE stained FBL-3 tumor cells and varying numbers of isolated NK cells from spleen of naive or FV-infected mice by MACS technology (Miltenyi Biotec). The cytotoxic assay was performed in 96-well U-bottom plates. The cells were co-incubated for 24 hours in a humidified 5% CO_2_ atmosphere at 37°C. Cells were washed once, resuspended in buffer containing 7-aminoactinomycin D to exclude dead cells and analyzed by flow cytometry.

### *In vivo* cytotoxicity assay

The *in vivo* cytotoxicity assay was performed as previously described [28]. Labeled target cells were transferred into naive or FV-infected TLR3^+/+^ or TLR3^−/−^ mice at day 10 post infection.

### Statistical analyses

Statistical analyses and graphical presentations were computed with Graph Pad Prism version 6. Two-group comparisons were done using 2-tailed unpaired Student’s t test. Analyses including several groups were tested using Kruskal-Wallis one-way analysis of variance on ranks and Dunn’s multiple comparison test (non-parametric distribution) or the ordinary one-way ANOVA and Bonferroni's multiple comparisons test (parametric distribution). P values less than 0.05 were considered statistically significant.

## Abbreviations

cGAS: Cyclic GMP-AMP synthase
FV: Friend Retrovirus
MDA5: Melanoma Differentiation-Associated protein 5
mDC: Myeloid dendritic cell
MyD88: Myeloid differentiation primary response 88
NK cell: Natural killer cell
PRR: Pattern recognition receptor
RIG-I: Retinoic acid inducible gene I
TLR: Toll-like receptor
